# “Green” Nanotechnologies: Synthesis of Metal Nanoparticles Using Plants

**Published:** 2014

**Authors:** V. V. Makarov, A. J. Love, O. V. Sinitsyna, S. S. Makarova, I. V. Yaminsky, M. E. Taliansky, N. O. Kalinina

**Affiliations:** Belozersky Institute of Physico-Chemical Biology, Lomonosov Moscow State University, Leninskie Gory 1, Bldg. 40, 119991, Moscow, Russia; Advanced Technologies Center, 4-5-47 Stroiteley Str., 119311, Moscow, Russia; The James Hutton Institute, Invergowrie, Dundee, DD2 5DA, Scotland, UK; Department of Physics, Lomonosov Moscow State University, Leninskie Gory 1, Bldg. 2, 119991, Moscow, Russia; Department of Biology, Lomonosov Moscow State University, Leninskie Gory 1, Bldg. 12, 119991, Moscow, Russia; Nesmeyanov Institute of Organoelement Compounds, Russian Academy of Sciences, Vavilova Str. 28, 119991, Moscow, Russia

**Keywords:** biomatrices, bioreduction, metal nanoparticles, plant metabolites, plant extracts

## Abstract

While metal nanoparticles are being increasingly used in many sectors of the economy, there is growing interest in the biological and environmental safety of their production. The main methods for nanoparticle production are chemical and physical approaches that are often costly and potentially harmful to the environment. The present review is devoted to the possibility of metal nanoparticle synthesis using plant extracts. This approach has been actively pursued in recent years as an alternative, efficient, inexpensive, and environmentally safe method for producing nanoparticles with specified properties. This review provides a detailed analysis of the various factors affecting the morphology, size, and yield of metal nanoparticles. The main focus is on the role of the natural plant biomolecules involved in the bioreduction of metal salts during the nanoparticle synthesis. Examples of effective use of exogenous biomatrices (peptides, proteins, and viral particles) to obtain nanoparticles in plant extracts are discussed.

## INTRODUCTION

The widespread practical application of metal nanoparticles (particles less than 100 nm) is attributable to a number of their unique properties [1-4]. Different physical and chemical processes are currently widely used to synthesize metal nanoparticles, which allow one to obtain particles with the desired characteristics [5-8]. However, these production methods are usually expensive, labor-intensive, and are potentially hazardous to the environment and living organisms [9, 10]. Thus, there is an obvious need for an alternative, cost-effective and at the same time safe and environmentally sound method of nanoparticle production [11-13]. During the past decade, it has been demonstrated that many biological systems, including plants and algae [14], diatoms [15, 16], bacteria [17], yeast [18], fungi [19], and human cells [20] can transform inorganic metal ions into metal nanoparticles via the reductive capacities of the proteins and metabolites present in these organisms. It is significant that the nanoparticle production using plants described in the present review displays important advantages over other biological systems. The low cost of cultivation, short production time, safety, and the ability to up production volumes make plants an attractive platform for nanoparticle synthesis [21].

## PLANTS AS BIOREACTORS FOR THE SYNTHESIS OF METAL NANOPARTICLE

It has long been known that plants are able to reduce metal ions both on their surface and in various organs and tissues remote from the ion penetration site. In this regard, plants (especially those which have very strong metal ion hyperaccumulating and reductive capacity) have been used for extracting precious metals from land which would be economically unjustifiable to mine; an approach known as phytomining. The metals accumulated by the plants can be recovered after harvesting via sintering and smelting methods. Interestingly, study of the metal bioaccumulation process in plants has revealed that metals are usually deposited in the form of nanoparticles. For example, *Brassica juncea* (mustard greens) and *Medicago sativa *(alfalfa) accumulate 50 nm silver nanoparticles to a high level (13.6% of their own weight) when grown on silver nitrate as a substrate [22]. In addition, gold icosahedra of 4 nm in size were detected in *M. sativa *[23], and semi-spherical copper particles with a size of 2 nm were observed in* Iris pseudacorus *(yellow iris) [24] grown on substrates containing salts of the respective metals. Whole plants can obviously be used to produce metal nanoparticles. However, there exists certain limitations that should be taken into account upon industrial application of this technology. Firstly, the size and shape of nanoparticles vary depending on their localization in the plant, which may depend on differences in the content of metal ions in various tissues and the subsequent possibility of nanoparticle movement and penetration. These factors could influence the level of metal deposition around already existing nanoparticles, and also the prospect of new nucleation events (initiation of nanoparticle formation) [23]. The heterogeneity of the size and morphology of nanoparticles produced in whole plants may hinder their use in applications where specific, finely tuned sizes and shapes are required; thus illustrating the inability to tailor the whole plant synthesized nanoparticles to market requirements. Moreover, efficient extraction, isolation and purification of nanoparticles from plant material is a difficult and problematic procedure, with a low recovery.

In this regard, *in vitro *approaches have actively been developed in recent years, in which plant extracts are used for the bioreduction of metal ions to form nanoparticles. These approaches provide a more flexible control over the size and shape of the nanoparticles (for example, by changing the medium pH and reaction temperature), as well as facilitating easy purification. Significantly, this process occurs much faster than the synthesis of nanoparticles in whole plants, because the reaction proceeds almost instantaneously, without the delay required for the uptake and diffusion of metal ions throughout the plant. This *in vitro *approach has been demonstrated using extracts from a variety of different plant species in combination with a variety of acids and salts of metals, such as copper, gold, silver, platinum, iron, and many others [22, 25-27].

**Fig. 1:**
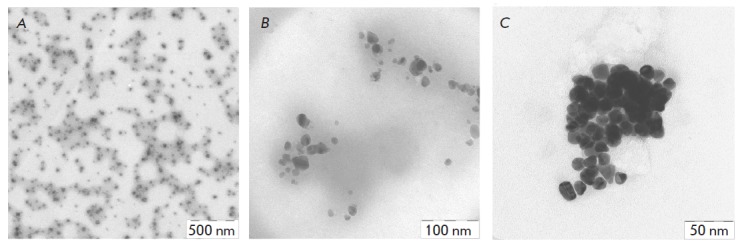
Electron micrographs of the iron (A), silver (B), and gold (C) nanoparticles synthesized in extracts of *N. benthamiana* at room temperature

For example, extracts of *Pelargonium graveolens* (rose geranium) have been used to reduce gold ions into 20–40 nm decahedral icosahedral shaped nanoparticles and stabilize them [28], whereas gold nanospheres and nanotriangles 0.05–18 μm in size have been synthesized in extracts from *Cymbopogon flexuosus* (lemon grass) [29]. The *Azadirachta indica *(neem, Indian lilac) extract was used to reduce tetrachloroauric acid (HAuCl4) to flat gold triangles and hexagons with a size of 50–100 nm [30]. In that study, it was also demonstrated that the *A. indica *juice can reduce silver nitrate to polydispersed spherical nanoparticles with a size of 5–25 nm [30]. The leaf extract of *Aloe barbadensis *(aloe vera) was used to produce cubic In2O3 particles 5–50 nm in size [31]. It has been demonstrated using FTIR spectroscopy that plant metabolites such as sugars, terpenoids, polyphenols, alkaloids, phenolic acids, and proteins play an important role in the reduction of metal ions into nanoparticles and in supporting their subsequent stability [29, 30, 32-34]. It has been suggested that control over the size and morphology of nanostructures may be connected to the interaction of these biomolecules with metal ions [30]. Various plants differ in the concentration and composition of these biologically active components. This may partly explain the morphological diversity of the described nanoparticles: triangles, hexagons, pentagons, cubes, spheres, ellipsoids, nanowires, and nanorods. The diversity in the morphology and size of nanoparticles synthesized from a variety of metal ions in extracts of various plants has been described in detail in the reviews [11, 35]. As an example, *Fig. 1* shows images of the iron, silver and gold nanoparticles produced in *Nicotiana benthamiana *extracts.

## THE ROLE OF PLANT METABOLITES IN THE BINDING AND REDUCTION OF METAL IONS

**Fig. 2:**
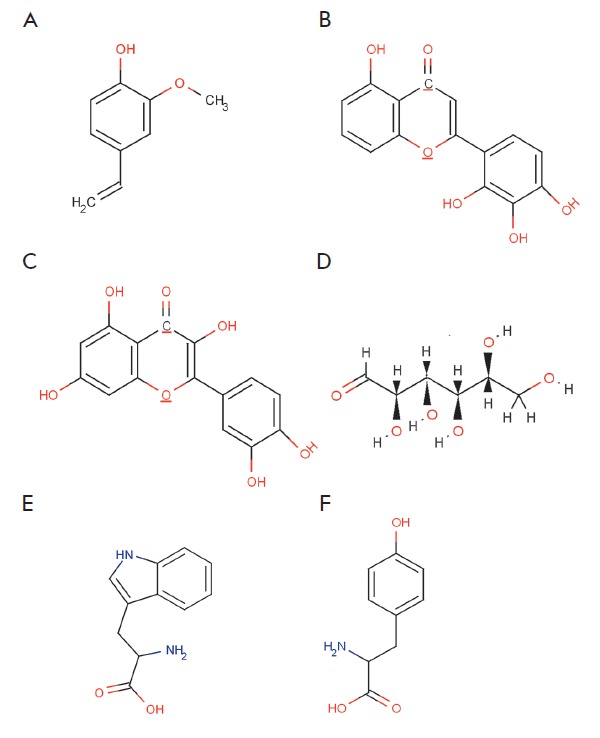
The main types of plant metabolites involved in the synthesis of metal nanoparticles: A – terpenoids (eugenol); B,C – flavonoids (luteolin, quertcetin); D – a reducing hexose with the open chain form; E,F – amino acids (tryptophan (E) and tyrosine (F))

As mentioned above, various plant metabolites, including terpenoids, polyphenols, sugars, alkaloids, phenolic acids, and proteins, play an important role in the bioreduction of metal ions, yielding nanoparticles. Examples of the main types of compounds capable of reducing metal ions are shown in *Fig. 2*.

Using FTIR spectroscopy of nanoparticles synthesized in plants/plant extracts, it has been demonstrated that terpenoids are often associated with nanoparticles. Terpenoids are a class of diverse organic polymers synthesized in plants from five-carbon isoprene units, which display strong antioxidant activity. Shankar *et al*. [29] initially suggested that terpenoids play a key role in the transformation of silver ions into nanoparticles in reactions using extracts from geranium leaves. Eugenol, the main terpenoid of *Cinnamomum zeylanisum* (cinnamon) extracts, was found to play the principal role in the bioreduction of HAuCl_4_ and AgNO_3_ to nanoparticles [36]. Based on the FTIR spectroscopy data, it was suggested [36] that dissociation of a proton of the eugenol OH-group results in the formation of resonance structures capable of further oxidation. This process is accompanied by the active reduction of metal ions, followed by nanoparticle formation.

Flavonoids are a large group of polyphenolic compounds that comprise several classes: anthocyanins, isoflavonoids, flavonols, chalcones, flavones, and flavanones, which can actively chelate and reduce metal ions into nanoparticles. Flavonoids contain various functional groups capable of nanoparticle formation. It has been postulated that the tautomeric transformations of flavonoids from the enol-form to the keto-form may release a reactive hydrogen atom that can reduce metal ions to form nanoparticles. For example, it is believed that in the case of *Ocimum basilicum *(sweet basil) extracts it is the transformation of flavonoids luteolin and rosmarinic acid from the enol- to the keto-form that plays a key role in the formation of silver nanoparticles from Ag ions [37]. Moreover, the internal mechanism of the conversion of ketones to carboxylic acids in flavonoids is likely to be involved in Au_3_^+^ ion reduction. Interestingly, some flavonoids are able to chelate metal ions with their carbonyl groups or π-electrons. For example, quercetin is a flavonoid with very strong chelating activity, because it can chelate at three positions involving the carbonyl and hydroxyls at the C3 and C5 positions and the catechol group at the C3’ and C4’ site. These groups chelate various metal ions such as Fe^2+^, Fe^3+^, Cu^2+^, Zn^2+^, Al^3+^, Cr^3+^, Pb^2+^, and Co^2+^. The presence of such mechanisms may indeed explain the ability of flavonoids to be adsorbed onto the surface of a nascent nanoparticle. This probably means that they are involved in the stages of initiation of nanoparticle formation (nucleation) and further aggregation, in addition to the bioreduction stage. Moreover, isolated flavonoids and flavonoid glycosides have the ability to induce the formation of metal nanoparticles. For example, apiin (apigenin glycoside) was extracted from *Lawsonia inermis *(lawsonite thornless, henna) and used for the synthesis of anisotropic gold and quasi-spherical silver nanoparticles with an average size of 21–30 nm [38]. A FTIR analysis revealed that apiin was attached to the nanoparticles through a carbonyl group.

There are data according to which the sugars present in plant extracts can also induce the formation of metal nanoparticles. It is known that monosaccharides such as glucose (linear and containing an aldehyde group) can act as reducing agents. Monosaccharides containing a keto-group, e.g. fructose, can act as antioxidants only when they have undergone a series of tautomeric transformations from a ketone to an aldehyde. Moreover, the reducing ability of disaccharides and polysaccharides depends on the ability of any of their individual monosaccharide components to adopt an open chain form within an oligomer and, hence, to provide access (of a metal ion) to an aldehyde group. For example, the disaccharides maltose and lactose have reducing ability, since at least one of their monomers can assume an open chain form. Sucrose, in contrast, has no ability to reduce metal ions, because glucose and fructose monomers are linked in such a way that the open chain form is not available. It was found [39] that glucose is capable of participating in the synthesis of metal nanoparticles of various morphologies, whereas fructose mediates the synthesis of monodispersed nanoparticles of gold and silver. Glucose was also noted [39] to be a stronger reducing agent than fructose, because the antioxidant potential of fructose is limited by the kinetics of tautomeric shifts (as discussed above). It was shown [39] that sucrose is unable to reduce silver nitrate or palladium chloride into nanoparticles. However, when these metal salts were replaced by tetrachloroauric and tetrachloroplatinic acids, nanoparticles were formed in the presence of sucrose, which is likely due to the acidic hydrolysis of sucrose into free glucose and fructose, which have an open chain-form structure. It is currently believed that the sugar aldehyde group is oxidized into a carboxyl group via the nucleophilic addition of OH-, which in turn leads to the reduction of metal ions and to the synthesis of nanoparticles. A similar mechanism was proposed for the bioreduction of gold ions using the magnolia vine extract [29].

FTIR analysis of nanoparticles synthesized in plants or plant extracts revealed that nascent nanoparticles are very frequently found in association with proteins [40]. Amino acids were found to differ in their ability to bind metal ions and to reduce them. For example, as Gruen observed [41], amino acids such as lysine, cysteine, arginine, and methionine are capable of binding silver ions. Other studies have shown that aspartate can reduce tetrachloroauric acid to form nanoparticles, although valine and lysine do not possess this ability [42]. Tan *et al*. [43] recently analyzed all of the 20 natural α-amino acids to determine their potential for reduction or binding of metal ions. They established that tryptophan is the strongest reducing agent for Au ions, whereas histidine is one of the strongest binding agents for Au ions. Amino acids can bind to metal ions through the amino and carbonyl groups of the main chain or through side chains, such as the carboxyl groups of aspartic and glutamic acid or a nitrogen atom of the imidazole ring of histidine. Other side chains binding metal ions include the thiol (cysteine), thioether (methionine), hydroxyl (serine, threonine, and tyrosine), and carbonyl groups (asparagine and glutamine) [44]. A study of the ability to reduce metal ions indicated that the hydroxyl groups of tyrosine and carbonyl groups of glutamine and asparagine are involved in the reduction process of Ag ions. Side thiol groups (e.g. of cysteine) and amino groups are also responsible for the reduction of metal ions.

After amino acids are linked to the peptide chain, their individual ability to bind and reduce metal ions may change. For example, the formation of the peptide backbone changes the functionality of the R-carbon of carboxylic acids and amines of some amino acid residues since they move to a form inaccessible for interaction with metal ions. However, free side chains of amino acids can still participate in the binding and reduction of metal ions. The suitability of side chains for this interaction may change depending on the amino acid sequence, which could affect the accessibility of individual groups. The work by Tan *et al*. [43] explained in detail how the amino acid sequence may affect the protein’s ability to chelate and/or reduce metal ions. It was found that synthesized peptides, composed of amino acids capable of effective binding of metal ions, and of amino acids possessing high reducing activity, had lower reduction parameters than expected. It was suggested that the strong sequestration of metal ions to the peptide was inhibitory to their subsequent reduction by reducing amino acids. It was also found that peptides containing amino acids that weakly bind metal ions such as leucine, phenylalanine, and proline were ineffective in reducing tetrachloroauric acid anions, probably because of their inability to retain metal ions close to the reduction sites. It was assumed [44] that protein molecules facilitating the formation of nanoparticles from metal ions display high reducing activity and high potential for attracting metal ions to the regions of a molecule that are responsible for reduction, but that their chelating activity is not excessive. The paper also demonstrated that the amino acid sequence of a protein can greatly affect the size, morphology, and amount of nascent nanoparticles. For example, a synthetic peptide GASLWWSEKL rapidly reduces metal ions to form a large amount of small nanoparticles less than 10 nm in size, whereas replacement of N-and C-terminal amino acid residues in a peptide (SEKLWWGASL) leads to a slower reduction reaction that results in the formation of larger nanospheres and nanotriangles about 40 nm in size. These data indicate that the peptides and proteins present in plant extracts probably play a very important role in determining the shape of nanoparticles and affect the overall yield of nanoparticles.

**Fig. 3:**
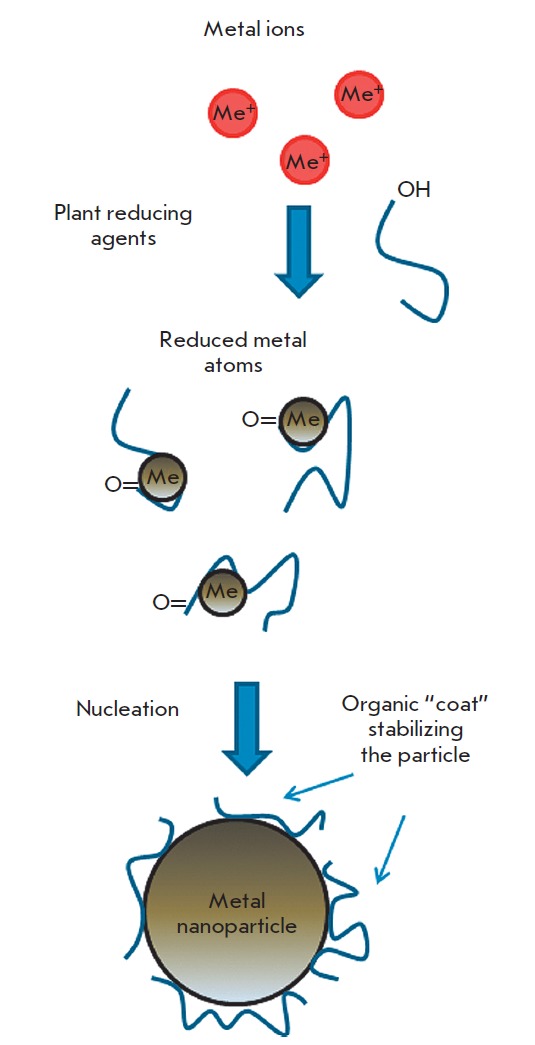
A schematic representation of metal nanoparticle synthesis in a plant extract. The metal ions bind to the reducing metabolites and stabilizing agents and are reduced to metal atoms. The resulting complex of the metal ion and metabolite interacts with similar complexes forming a small metal nanoparticle. Next, growth and coalescence of separate small particles into larger ones occur through the coarsening process. This process continues until the particles assume a stable shape and size

On the whole, the mechanism of metal nanoparticle synthesis in plants and plant extracts includes three main phases: 1) the activation phase during which the reduction of metal ions and nucleation of the reduced metal atoms occur; 2) the growth phase during which the small adjacent nanoparticles spontaneously coalesce into particles of a larger size (direct formation of nanoparticles by means of heterogeneous nucleation and growth, and further metal ion reduction; a process referred to as Ostwald ripening), which is accompanied by an increase in the thermodynamic stability of nanoparticles; and 3) the process termination phase determining the final shape of the nanoparticles [44, 45]. The process of nanoparticle formation is shown schematically in *Fig. 3*. As the duration of the growth phase increases, nanoparticles aggregate to form nanotubes, nanoprisms, nanohexahedrons, and a variety of other irregularly shaped nanoparticles, as well [44, 46]. In the termination phase, nanoparticles acquire the most energetically favorable conformation, with this process being strongly influenced by the ability of a plant extract to stabilize metal nanoparticles. For example, nanotriangles have a very high surface energy, which makes them less stable, and if the stability of nanoparticles is not supported in given extracts, then the nanotriangles will acquire a more stable morphology, such as a truncated triangle, in order to minimize the Gibbs free energy.

## OTHER FACTORS AFFECTING THE FORMATION OF METAL NANOPARTICLES IN PLANTS

Therefore, the reduction process of metal ions with the formation of nanoparticles is affected by a large number of factors; besides the nature of a plant extract containing active biomolecules in different combinations and concentrations (the effects of which are described above), these include the reaction mixture pH, incubation temperature, reaction time, concentration, and electrochemical potential of a metal ion [11, 35, 47, 48].

The pH value of a plant extract exerts great influence on the formation of nanoparticles [49-52]. A change in pH results in a charge change in the natural phytochemicals contained in an extract, which affects their ability to bind and reduce metal cations and anions in the course of nanoparticle synthesis, and this in turn may affect the shape, size, and yield of nanoparticles. For example, in the *Avena sativa *(common oat) extract more numerous small-sized gold nanoparticles were formed at pH 3.0 and 4.0, whereas more aggregated particles were observed at pH 2.0. Therefore, it has been suggested that nanoparticle aggregation is dominant over the process of reduction and primary nucleation of reduced atoms at very acidic pH values. This may perhaps be related to the fact that a larger number of functional groups that bind and nucleate tetrachloroauric acid ions become accessible at pH 3.0 and 4.0 than at pH 2.0. At pH 2.0 the most accessible metal ions are apparently involved in a smaller number of nucleation events, which leads to agglomeration of the metal [52]. In contrast, it was demonstrated using extracts from pears that hexagonal and triangular gold nanoplates are formed at alkaline pH values, whereas nanoparticles do not form at acidic pHs [50]. In the case of silver ions (1+) and the tuber powder of *Curcuma longa *(turmeric), a substantially larger number of silver nanoparticles are synthesized at alkaline pHs, at which extracts may contain more negatively charged functional groups capable of efficient binding and reduction of silver ions [52].

Temperature is another important factor affecting the formation of nanoparticles in plant extracts [53-57]. In general, temperature elevation increases the reaction rate and efficiency of nanoparticle synthesis. It was found that in alfalfa plants (*M. sativa*) triangular silver nanoparticles formed only at temperatures above 30 °C [54]. Furthermore, experiments on the synthesis of silver nanoparticles in lemon verbena extracts (*Aloysia citrodora*) demonstrated that increasing the reaction temperature is accompanied by an increase in the efficiency of the silver ion reduction [56]. Moreover, crystal particles are formed much more frequently at high temperatures than at room temperature. It is assumed that elevating the temperature increases the nucleation rate. In experiments with *Cassia fistula *(golden shower tree) extracts, it was found that temperature may also affect the structural form of the synthesized nanoparticles; silver nanoribbons are mainly formed at room temperature, whereas spherical nanoparticles predominate at temperatures above 60 °C [55]. In this case it is believed that higher temperatures alter the interaction of phytochemicals with the nanoparticle surface, thereby inhibiting incorporation of adjacent nanoparticles into the structure of nanoribbons. Furthermore, in some situations higher temperatures may facilitate the nucleation process to the detriment of the secondary reduction process and further condensation of a metal on the surface of nascent nanoparticles. This phenomenon is believed to explain the formation of the spherical gold nanoparticles in *Nyctanthes arbortristis* (jasmine night) alcoholic extracts at 80 °C in contrast to the nanoparticles of different morphology and “nanocolors” formed at room temperature [57].

Due to the limited ability of plants to reduce metal ions, the efficiency of metal nanoparticle synthesis also depends on the electrochemical potential of an ion [35]. Thus, the ability of a plant extract to effectively reduce metal ions may be significantly higher in the case of ions having a large positive electrochemical potential (for example, Ag^+^) than in the case of ions with a low electrochemical potential such as ([Ag(S_2_O_3_)_2_]^3-^) [35].

As discussed above, the proteins that are present in a plant extract may significantly affect the formation of nanoparticles. The approaches that have recently been used for the “green” synthesis of metal nanoparticles combine the use of plant extracts with the exogenous supplementation of the *in vitro *reactions with biomatrices: peptides, and proteins, whose amino acid sequence and structure are optimized for the efficient production of nanoparticles. The phage display method is used for the search for peptides with the appropriate characteristics. Tryptophan and amino acids such as tyrosine, arginine, and lysine possess superior ability to reduce metal ions. However, a polypeptide composed only of tryptophan residues is much less effective than a mixture of tryptophan molecules interspersed with other amino acids at forming nanoparticles, likely due to strong binding of the reduced ion, which in turn is inhibitory to further reduction. In turn, peptides that consist of different amino acids (for example, RWRWRWRWR) capable of strongly binding metal ions are also poorly suited as a biomatrix for the synthesis of nanoparticles due to entropic effects. Peptides comprising amino acids that weakly bind tetrachloroauric acid ions, such as glutamic or aspartic acids, are also inefficient in the synthesis of nanoparticles because of rapid dissociation of the peptide - metal ion complex. Therefore, the most suitable peptides for the formation of metal nanoparticles are those in which reducing and strongly binding amino acid residues (e.g., tryptophan) alternate with weakly binding amino acids that act as an up-regulator. The next important stage in the formation of metal nanoparticles is the agglomeration of reduced metal atoms to nanoparticles. This process depends on many factors and determines a number of the properties of a nascent nanoparticle such as its size or shape. At the beginning of the process, small particles of the reduced metal are formed on the surface of a peptide biomatrix that are further redistributed into larger nanoparticles during Ostwald ripening (coarsening). It has been shown that the faster and more efficient nanoparticle formation is, the more isotropic and smaller nanoparticles are produced, since the coarsening effectiveness is time-dependent. For example, it was possible to vary the shape and size of produced nanoparticles by altering the amino acid sequence and thus the reaction kinetics of nanoparticle formation.

**Fig. 4:**
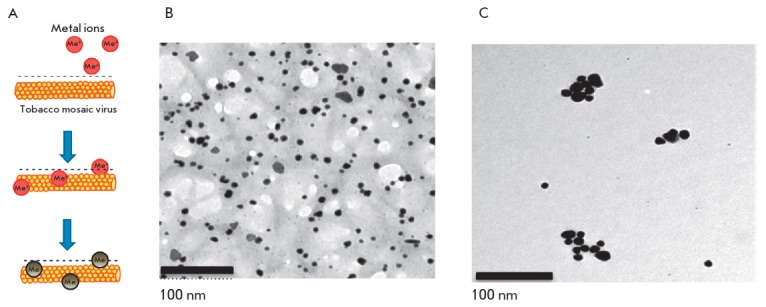
Formation of nanoparticles in plant extracts using biomatrices. A **– **A scheme of nanoparticle formation in the presence of the tobacco mosaic virus (TMV) as a biomatrix. Metal ions interacting with negatively charged groups on the TMV surface are reduced upon addition of a plant extract. The regular positioning of TMV active groups significantly increases the number of effective events of initiation, which increases the output of metal nanoparticles 3–5-fold. B,C – electron micrographs of gold nanoparticles produced in *N. benthamiana *extracts in the presence (B) and in the absence (C) of TMV particles

**Fig. 5:**
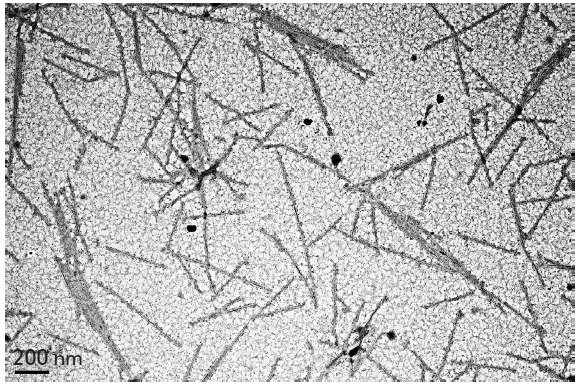
Micrograph of the metallized tobacco mosaic virus obtained by transmission electron microscopy

The identified patterns also hold for macromolecular biomatrices, such as viruses and virus-like particles. In one of the first studies carried out using this approach, a plant RN A virus with helical symmetry (tobacco mosaic virus (TMV) with a length of 300 nm and a width of 18 nm) or icosahedral particles of the non-infectious bovine papilloma virus (particles with a diameter of 55 nm obtained by self-assembly of the viral envelope protein expressed in plants) were added to silver or gold salts before adding *N. benthamiana *or barley extracts [58, 59]. In the presence of virus/viruslike particles at low concentrations, a decrease in the size of nanoparticles and a 4- to 5-fold increase in their number was observed compared to samples containing no virus (*Fig. 4 B,C*) [58,59]. Interestingly, the amount of formed nanoparticles was significantly less at a high TMV concentration, but viral particles were metallized (*Fig. 5*). Amino acid side chains capable of chelating and reducing metal ions are exposed on the inner and outer surfaces of the TMV and on the outer surface of bovine papilloma virus particles. For example, carboxyl and hydroxyl groups are accessible on the TMV outer surface as part of the exposed peptide segments of the surface loop and C-terminal region, while amino acid amino groups are accessible in the TMV inner channel [60]. Carbonyl, hydroxyl, and carboxyl groups are accessible on the surface of bovine papilloma virus particles [58]. Another important factor allowing viruses and virus-like particles to be considered as effective biomatrices for nanoparticle synthesis is the mutual spatial orientation of active peptide groups due to the structural regularity of these biomatrices. This can lead to significant acceleration of the synthesis reaction and, consequently, to an increasing yield of nanoparticles. The surface of a viral particle consists of dense arrays of appropriately packed capsid proteins (a single TMV particle consists of 2,130 molecules of the capsid protein and a bovine papilloma virus particle consists of 360 molecules of the capsid protein) that form a highly reactive quasiregular surface capable of interacting with metal ions. At low virus concentrations in a reaction mixture, metal ions are believed to react with amino acid residues in the molecules of the viral capsid protein that initiates the nucleation process. Preformed nucleation centers undergo a significantly more rapid reduction upon addition of a plant extract. The presence of a virus in a reaction mixture probably inhibits aggregation by increasing the number of nucleation sites, which reduces the accumulation of metal ions around a smaller number of points. At increased virus concentrations, the elevated number and concentration of clustered active amino acid residues in a small space enhances the binding and reduction of metal ions in the vicinity of the virus surface; effectively decreasing the development of free nucleation centers and inhibition of metal nanoparticle formation outside viral clusters (“free” nanoparticles). Subsequent addition of plant extracts quickly reduces metal ions chelated by viral particles, which leads to metallization of viral particles (e.g., to the formation of nanowires in the case of TMV) but decreased rate of formation of “free” nanoparticles.

## PROSPECTIVE APPLICATION OF NANOPARTICLES SYNTHESIZED IN PLANTS

The diversity of plant extracts and types of metal salts, the ability to alter the composition of a reaction mixture and reaction conditions through changes in the temperature, pH, and inclusion of additives of biological origin (biomatrices), allow one to produce nanoparticles of various metals with a defined size and shape. Despite the fact that “green” synthesis of nanoparticles using plant material is of considerable interest, it is worth studying the equivalence of these nanoparticles with nanoparticles produced through physical and chemical methods, especially with regard to their potential applications and production scalability. For example, it is well-known that traditional physicochemically synthesized metal nanoparticles have been used in cancer therapy, the targeted delivery of drugs, molecular imaging, wastewater treatment, catalysis, biosensor development, fuel elements, coatings, cosmetics and as antiseptics. Nanoparticles produced in plants/ plant extracts have been tested so far only in a small number of practical applications. For example, silver nanoparticles produced using the *Tridax procumbens* (tridax daisy) extract display, similar to their equivalents obtained using chemical or physical methods, strong antimicrobial activity against *Escherichia coli*,* Shigella dysenteriae*, and *Vibrio cholera *[61]. Silver nanoparticles obtained using *Pinus thunbergii *(Japanese black pine) cone extracts exhibit antibacterial activity against various Gram-positive and Gram-negative agricultural pathogens, such as *Pseudomonas syringae*,* Xanthomonas oryzae*, *Burkholderia glumae*, and *Bacillus thuringiensis *[62]. Silver nanoparticles synthesized in plants display significant cytotoxic activity against various tumor cell lines. Silver nanoparticles synthesized in *Iresine herbstii *(Herbst’s bloodleaf) were found to inhibit the survival and growth of HeLa cell lines, and silver nanoparticles produced using *Euphorbia nivulia *(leafy milk hedge) latex extracts are toxic to the A549 cell line of human lung cancer [63]. Silver nanoparticles synthesized in *Nerium oleander *(oleander) display strong larvicidal activity against larvae of the malaria vector *Anopheles stephensi *[64].

In perspective, functionalization of these particles with antibodies or peptides is intended for targeted action in certain tissues of the body in order to achieve greater efficiency and reduce side effects. *Cyamopsis tetragonoloba *(cluster bean) extracts were used recently to produce composite silver nanoparticles that can act as a biosensor to determine ammonia, with possible applications in agriculture and biomedicine. Depending on the ammonia concentration, the distance between the nanoparticles inside the nanocomposite changes, which affects its optical properties [65]. Platinum nanoparticles obtained using *Ocimum sanctum *(Holy basil) extracts were shown to possess a catalytic activity and may be used in the production of hydrogen fuel elements [66]. Catalytic activity is also ascribed to gold nanoparticles obtained in *Sesbania drummondii *(rattlebush) that may participate in the reduction of aromatic nitro compounds; for example, convert highly toxic 4-nitrophenol to 2-amino-phenol, which suggests their possible involvement in waste decontamination.

These examples are not an exhaustive list of all the data on the application of “green” nanoparticles: however, they provide strong evidence of a possible practical application of nanoparticles synthesized using plants/plant extracts. The main question that needs to be answered is whether the biological and physicochemical characteristics of nanoparticles of plant “origin” differ from the characteristics of their existing prototypes and to what extent these differences affect efficiency in nanoparticle application for specific practical problems. It is significant to note that nanoparticles synthesized in plant extracts already have a functionalized surface that can contain (depending on reaction conditions) the organic ligands, proteins, polysaccharides, and polyatomic alcohols that are absent in nanoparticles synthesized using physical and chemical methods. The presence of these biological components promotes, as is known, an increase in the stability of the particles and may also facilitate, if necessary, the subsequent attachment of functional molecules, such as antibodies or DNA, to nanoparticles [67].

## CONCLUSIONS

Obviously, the synthesis of metal nanoparticles in plant extracts (plant biomasses), despite obvious limitations, has a significant potential and a number of substantial advantages relative to traditional methods of nanoparticle synthesis. However, to compete cost-effectively with nanoparticles obtained through physical and chemical methods, it is necessary to scale these methods of nanoparticle production using plant material and to develop schemes for keeping expenses in check during their synthesis. Continuous methods for the synthesis of nanoparticles have so far been used only in small-scale production. When using chemical synthesis, the prime cost of nanoparticles is mainly determined by the cost of the metal salts and reducing agents. In the case of “green” synthesis, the bulk of the costs will be determined only by the cost of the metal salts, because plant wastes from the food industry can serve as reducing agents. Moreover, it is possible to envision companies involved in the food industry and interested in the recycling of waste to partially pay for nanoparticle production. This fact further emphasizes the environmental advantages of “green” synthesis over traditional methods of nanoparticle production.
